# Reviewed article: Cordeiro DC, Aguiar EB, Luiz RR, Sacramento DS, Paolino PLW, Gonçalves MJF, Trajman A. Effect of successive tuberculin skin test readings on healthcare professionals’ proficiency: an operational observational study in five high-tuberculosis-burden Brazilian municipalities, 2023-2024. Epidemiol Serv Saude. 2025:34;e20240795

**DOI:** 10.1590/S2237-96222025v34e20240795.a

**Published:** 2025-10-31

**Authors:** Paula Brustolin Xavier

**Affiliations:** 1 Universidade do Oeste de Santa Catarina, Joaçaba, SC, Brazil Universidade do Oeste de Santa Catarina Joaçaba SC Brazil

## Peer review A

## Questionnaire

Does the manuscript contain new and significant information to justify publication? Yes

Does the Abstract (Summary) clearly and accurately describe the content of the article? No

Is the problem significant and concisely stated? Yes

Are the methods described comprehensively? No

Are the interpretations and conclusions justified by the results? No

Is adequate reference made to other work in the field? Yes

## Manuscript Structure

Length of article is: Too short

Number of tables is: Adequate

Number of figures is: Adequate

Please state any conflict(s) of interest that you have in relation to the review of this paper. None

Interest: Good

Quality: Below Average

Originality: Good

Overall: Average

Recommendation: Minor Revision

## Comments

Prezados autores,

Segue algumas sugestões no intuído de tornar o artigo com maior teor de cientificidade e ser citado por outros pesquisadores cujo interesse seja o mesmo.

Título - Sugiro que para tornar o artigo mais atraente e instigante ao leitor, o título seja modificado sem perder a relevância do tema, porém destaque o problema central de forma mais clara

Ex: Eficácia na capacitação para a prova tuberculínica: Quantas leituras são realmente necessárias?

Resumo: Descrever o delineamento do estudo, programa estatístico utilizado, conclusão escrever de forma mais clara.

Introdução

Sugiro descrever um parágrafo sobre a doença (sinais, sintomas).

Ao revisar a metodologia, gostaria de sugerir algumas melhorias que podem ajudar a tornar a descrição mais clara, detalhada e alinhada com os padrões de redação científica. Seguem algumas sugestões:

Detalhamento das Etapas: Recomendo descrever cada etapa do estudo de forma sequencial e detalhada. Por exemplo, como os profissionais foram selecionados, quais critérios de inclusão e exclusão foram utilizados e como o treinamento foi conduzido.

Descrição do Instrumento: Seria importante fornecer mais detalhes sobre os instrumentos utilizados, como as leituras da prova tuberculínica foram realizadas (ferramentas, tempo entre leituras, se houve algum individuo ou não) e qualquer protocolo seguido (estudo inovador)

Reprodutibilidade: Certifique-se de que a metodologia contenha informações suficientes para que o estudo possa ser replicado por outros pesquisadores. Inclua informações método de análise de dados e softwares utilizados (se aplicável).

Justificativa para as Escolhas Metodológicas: Explique o motivo por trás de algumas escolhas metodológicas, como o número de leituras considerado ideal ou os critérios utilizados para avaliar a capacitação dos profissionais.

Descrição da Análise de Dados: Detalhe os métodos estatísticos ou analíticos empregados, incluindo os testes realizados, níveis de significância adotados e como os resultados foram interpretados.

Na discussão senti falta referências adicionais que possam corroborar os achados ou discutir métodos alternativos.

Incorporação de Estudos Relacionados: Considero importante incluir mais artigos que sustentem ou contrastem os resultados encontrados. Isso ajudará a contextualizar os achados do estudo em relação ao que já foi publicado na área.

Comparação com Métodos Alternativos: Seria interessante mencionar outras abordagens metodológicas utilizadas em estudos semelhantes e discutir como os resultados deste trabalho se alinham ou diferem dessas abordagens.

Exploração de Implicações Práticas: Sugiro expandir a discussão sobre as implicações práticas dos resultados, especialmente no contexto da capacitação para a prova tuberculínica. Como esses achados podem influenciar a prática ou o treinamento futuro? Além disso contextualizar com a sintomatologia e o quadro clínico da pessoa portadora do bacilo, muito embora seja assintomática, mas tem um histórico de doença familiar e ou contato.

Observei a ausência de uma seção de considerações finais ou conclusões. Essa seção é fundamental para sintetizar os principais achados do estudo, reforçar sua relevância e destacar as implicações práticas ou científicas. Sugiro incluir essa parte no texto, abordando pontos como:

Resumo dos Principais Achados: Reforce os resultados mais importantes e o que eles acrescentam ao conhecimento atual sobre a capacitação para a prova tuberculínica.

Implicações Práticas e Científicas: Destaque como os achados podem influenciar as práticas de treinamento ou políticas públicas relacionadas ao tema.

Recomendações Futuras: Apresente sugestões para estudos futuros ou possíveis melhorias em metodologias relacionadas.

Mensagem Final: Inclua uma frase de fechamento que sintetize a contribuição do estudo e inspire o leitor a refletir sobre o tema.

